# Clinical Workflow Algorithm for Preoperative Planning, Reduction and Stabilization of Complex Acetabular Fractures with the Support of Three-Dimensional Technologies

**DOI:** 10.3390/jcm13133891

**Published:** 2024-07-02

**Authors:** Arpad Solyom, Flaviu Moldovan, Liviu Moldovan, Gabriela Strnad, Pal Fodor

**Affiliations:** 1 Orthopedics—Traumatology Department, Faculty of Medicine, “George Emil Palade” University of Medicine, Pharmacy, Science, and Technology of Targu Mures, 540142 Targu Mures, Romania; arpad.solyom@umfst.ro (A.S.); pal.fodor@umfst.ro (P.F.); 2 Faculty of Engineering and Information Technology, “George Emil Palade” University of Medicine, Pharmacy, Science, and Technology of Targu Mures, 540142 Targu Mures, Romania; liviu.moldovan@umfst.ro (L.M.); gabriela.strnad@umfst.ro (G.S.)

**Keywords:** acetabular fractures, preoperative planning, three-dimensional technologies, clinical workflow

## Abstract

**Background:** Treatment of pelvic injuries poses serious problems for surgeons due to the difficulties of the associated injuries. The objective of this research is to create a clinical workflow that integrates three-dimensional technologies in preoperative planning and performing surgery for the reduction and stabilization of associated acetabular fractures. **Methods**: The research methodology consisted of integrating the stages of virtual preoperative planning, physical preoperative planning, and performing the surgical intervention in a newly developed clinical workflow. The proposed model was validated in practice in a pilot surgical intervention. **Results**: On a complex pelvic injury case of a patient with an associated both-column acetabular fracture (AO/OTA-62C1g), we presented the results obtained in the six stages of the clinical workflow: acquisition of three-dimensional (3D) images, creation of the virtual model of the pelvis, creation of the physical model of the pelvis, preoperative physical simulation, orthopedic surgery, and imaging validation of the intervention. The life-size 3D model was fabricated based on computed tomography imagistics. To create the virtual model, the images were imported into Invesalius (version 3.1.1, CTI, Brazil), after which they were processed with MeshLab (version 2023.12, ISTI—CNR, Italy) and FreeCAD (version 0.21.2, LGPL, FSF, Boston, MA, USA). The physical model was printed in 21 h and 37 min using Ultimaker Cura software (version 5.7.2), on an Ultimaker 2+ printing machine through a Fused Deposition Modeling process. Using the physical model, osteosynthesis plate dimensions and fixation screw trajectories were tested to reduce the risk of neurovascular injury, after which they were adjusted and resterilized, which enhanced preoperative decision-making. **Conclusions**: The life-size physical model improved anatomical appreciation and preoperative planning, enabling accurate surgical simulation. The tools created demonstrated remarkable accuracy and cost-effectiveness that support the advancement and efficiency of clinical practice.

## 1. Introduction

Pelvic injuries are known to be among the most difficult injuries to treat. They are often the result of high-energy trauma and can lead to significant morbidity [1]. In the case of minimally displaced fractures where the loss of stability is limited, orthopedic treatment and management consist of conservative methods. Factors influencing the decision for surgical treatment of acetabular fractures are both fracture-related and patient-related. Some of the well-known fracture-related criteria are the biomechanical stability (based on Letournel classification fracture type), the comminution grade, and the displacement grade (usually fractures that have a roof-arc angle of <40°). In contrast, patient-related factors such as age, gender, and associated comorbidities are not as well defined [2]. To determine the optimal management of acetabular fractures, Heiman et al. [3] show that further research is needed.

A preoperative computed tomography (CT) scan is mandatory in all pelvic injuries to allow accurate planning of the surgical approach and technique. Intraoperatively, the surgeon can usually obtain standard two-dimensional (2D) views of the pelvic ring and acetabulum. Because of the anatomy and overlapping structures, evaluation of 2D views may yield limited information. For this reason, intraoperative three-dimensional (3D) imaging has become common in the last decade. The radiographic evaluation can also be affected by the orthopedic implants used. This disadvantage is limited by intraoperative CT imaging, which provides improved image quality and allows a considerably expanded field of view [4].

In the general population, variations in anatomical shape are largely related to differences in pelvic size [5]. Therefore, starting from the finding that the pelvis has considerable bilateral symmetry, unilateral pelvic fractures can be accurately reconstructed [6]. Thus, the contralateral hemipelvis can be used as a template for the reconstruction of the fractured hemipelvis. But this technique can be used for fractures with displacements that average below 2 mm root mean square (RMS) difference on the 3D deviation analysis [7]. The described technique cannot be applied to bilateral pelvic fractures. Ead et al. [8] report statistical modeling for the construction of pelvic mean shapes that can be used as templates for bilaterally fractured pelvis reconstruction. In preoperative planning, the reduced position of the fractured pelvis can be constructed with the aid of digital reduction algorithms created with the support of statistical shape models [9]. However, Handrich et al. [10] indicate that the pelvic ring has been shown not to be completely symmetrical, and lateral differences in size, shape and/or position must be considered. They studied 150 pelvic 3D CT models and found differences in terms of asymmetry of the pelvic ring related to gender and race, emphasizing preoperative planning in terms of biomechanical considerations, functional behavior and implant development.

In patients with pelvic trauma requiring secondary interventions, the mismatch of complex geometries between the pelvis and the fixation implant is a fundamental cause of disruption of the unstable and displaced pelvic ring [11]. Percutaneous fixation has become increasingly common for both the anterior and posterior ring and utilizes the placement of screws in the bony fixation pathways of the pelvis [12,13]. Robot-assisted surgery offers a new modality for the minimally invasive treatment of pelvic ring injuries, but it only allows for one-time screw placement due to the pre-planned positions that help reduce the deviation of the needle path and needle penetration [14].

Severe pelvic fractures often prove challenging for surgeons as they require patient-specific surgical treatment plans and customized equipment. The development of patient-specific virtual 3D pelvis models would facilitate the surgical planning process and enable the development of customized fixation plates [15,16,17]. For unstable pelvic ring fractures, 3D-printed custom osteosynthesis plates offer an improved treatment option. This is not adequately ensured by using the standard components available. The design of osteosynthesis plates that fix bone fragments as they were located before trauma requires the reduction and reconstruction of fractures with the support of medical imaging. This is a slow process that requires segmentation and repositioning of fragments until fracture surface congruences are obtained [18].

However, custom 3D-printed plates with virtual reduction model support favor easy and precise reductions in pelvic bone fractures [19]. Therefore, compared to open reduction and conventional internal fixation, the 3D-printing-assisted technique should be a more appropriate treatment of pelvic fractures [20,21]. Its main advantages are related to shorter operative time, less intraoperative blood loss and a lower complication rate. Limitations arise when printing intricate details of the human body as well as replicating small specimens [22,23]. However, research shows that the two techniques do not differ significantly as assessed by the high rates of both the Matta reduction score and the hip function score [24].

Three-dimensional printing refers to a range of manufacturing technologies that generate a physical model from digital information [25]. The spread of the application of 3D technology is supported by the added functionality of 3D polymer nanocomposites [26]. With this support, it offers expanded creativity in medicine and increases the utility of medical imaging due to its suitability for anatomical modeling [27]. At the same time, diagnostic imaging modalities play a fundamental role in the creation of 3D-printed models [28]. This new technology is also expanding into orthopedics due to its potential in surgical planning, orthotics, and manufacturing custom implants and guides, and for its implications in precision and personalized medicine [29,30,31].

Having as a starting point these controversies revealed by the scientific literature regarding the reduction and stabilization of associated acetabular fractures, in the present study, we formulated the following research questions:

RQ1: What are the possibilities of integrating three-dimensional technologies into the current practice of preoperative planning of complex orthopedic surgeries?

RQ 2: What is the content of the clinical workflow for preoperative planning with the support of three-dimensional technologies in the reduction and stabilization of associated acetabular fractures?

With the support of these research questions, we proposed the objective of our study to develop and test the efficiency and integrability in current medical practice of a clinical workflow for preoperative planning and performing the surgical intervention in the reduction and stabilization of complex acetabular fractures with the support of 3D printing technologies.

## 2. Materials and Methods

In the research methodology, we first developed a route of the surgical intervention that includes the stages of preoperative planning, performing the surgical intervention and its pilot validation in current medical practice. For this, we went through the following steps:

(2.1) We developed a clinical workflow for preoperative planning and surgical intervention in the reduction and stabilization of pelvic fractures with the support of three-dimensional technologies.

(2.2) We have designed the steps of virtual preoperative planning.

(2.3) We designed the stages of physical preoperative planning.

(2.4) We validated in practice the developed theoretical model by performing a pilot surgical intervention and postoperative imaging validation.

Preoperative processes are carried out in stages (2.1), (2.2), and (2.3), and intraoperative processes are carried out in stage (2.4), which also contains postoperative processes.

### 2.1. Clinical Workflow for Preoperative Planning and Surgical Intervention

**Figure 1:**
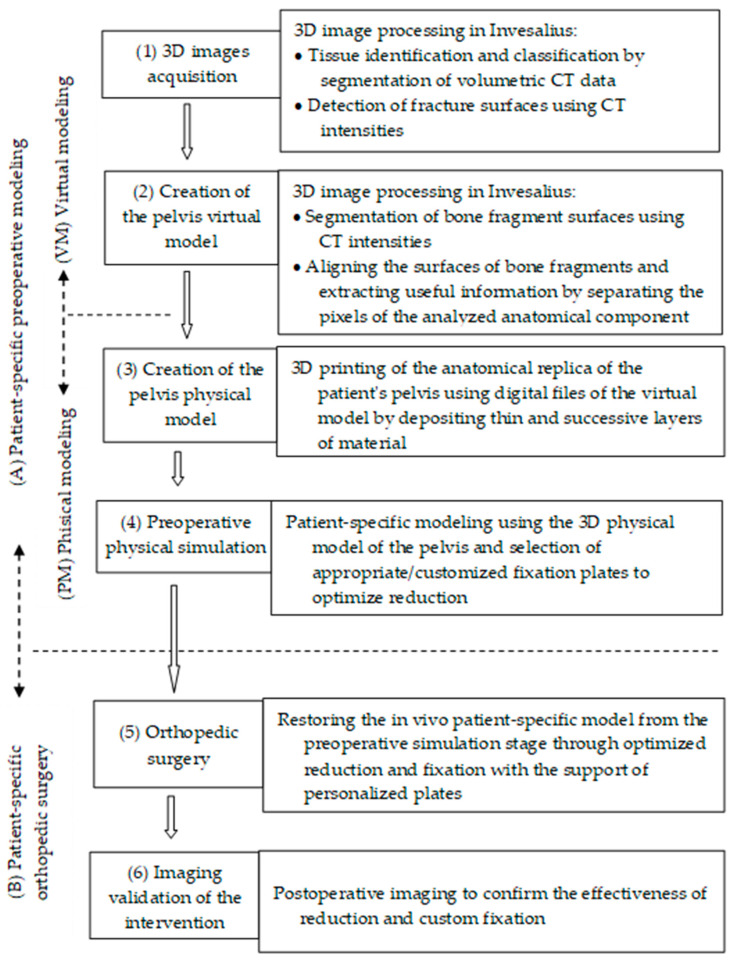
Clinical workflow integrating 3D printing technologies in preoperative planning and performing surgery for the reduction and stabilization of pelvic fractures: (1)–(4) are preoperative processes; (5) is an intraoperative process; (6) is a postoperative process.

By integrating 3D printing technologies, we developed a clinical workflow for the surgical intervention to reduce and stabilize acetabular fractures (Figure 1). It contains six stages grouped into two distinct periods. The first period is the preoperative one (A), patient-specific preoperative modeling, which is designed to take place immediately after the initial evaluation of the patient in the emergency room by respecting the early appropriate care (EAC) protocol. The aim is to first achieve rapid stabilization, both hemodynamically and of the fracture. These are key points that allow for early total care (ETC) with appropriate damage control (DCO), planning of the appropriate fracture fixation and reachable physiological parameters within the desirable 36 h. The second period is the intraoperative and postoperative period (B), patient-specific orthopedic surgery, which, although variable to each patient due to the particularities of the case, is optimized to limit the second hit and operative resuscitation.

The preoperative period (A) consists of two subperiods. The first of these is Virtual Modeling (VM), which encompasses the following steps: (1) 3D image acquisition, and (2) creation of the virtual pelvis model. The second preoperative subperiod is Physical Modeling (PM), which combines the following two steps: (3) creation of the pelvis physical model and (4) preoperative physical simulation.

Intraoperative and postoperative period (B)—patient-specific orthopedic surgery consists of the stages (5) orthopedic surgery and (6) imaging validation of the intervention.

### 2.2. Virtual Preoperative Planning

The new generation of three-dimensional (3D) technologies consists of imaging, digital design, numerical simulation, and rapid prototyping, which allow processing with different software products that analyze tissues, extract specific information, and have the potential to make medical investigations more efficient [32]. These technologies constitute a package of 3D tools that can be embedded in orthopedics which enable efficient diagnosis, more accurate preoperative planning, and the creation of customized surgical instruments, surgical guides, and patient-specific implants [33].

In cases of complex pelvic fractures, a critical factor is the restoration of bone morphology from its fragments. For this, algorithms are needed that align the surfaces of comminuted fractures. Their application is supported by the structured integration of new 3D technologies. For this purpose, we created a clinical workflow integrating 3D technologies, which aims to apply the alignment algorithm for the personalized surgical treatment of pelvic fractures (Figure 1) [34]. It is structured in six stages.

In stage (1), 3D image acquisition, the patient’s CT/MRI imaging is processed in Invesalius in two steps. The first step, tissue identification and classification by segmentation of volumetric CT data, generates surfaces of bone fragments that can be subsequently aligned. Identifying the location of fracture surfaces is a challenge because it is carried out with the support of points located on different surfaces. These are characterized by noises, which are quantified by Hounsfield intensities. In the second step, with the support of CT intensities, a covering surface is generated for each segmented bone. After that, pairs of points on the fractured surfaces are identified. We found that the cortical bone tissue has CT densities in the range of 700–2500 Hounsfield units, and the spongy tissue has intensities of approximately 700 Hounsfield units. The pelvis can be characterized as a sandwich-type structure formed mainly of cancellous bone embedded in a thin covering of cortical bone. Therefore, we deduced that most of the fractures in the pelvic region come from bone with low density. With the support of Hounsfield intensities, we managed to distinguish the points located on the fractured surface from the points located on the intact surface.

Stage (2), creation of the virtual pelvis model, continues with the digital image processing in two steps with the support of Invesalius. In the first step, the segmentation of the surfaces of the bone fragments takes place using the CT scales. We segment all the analyzed surfaces and with the support of Hounsfield intensities, we separate them into fracture surfaces and intact bone surfaces. Thus, we generate probable pairs of bone surfaces, but which do not match perfectly. In the second step, the surfaces of the bone fragments are aligned, and useful information is extracted by separating the pixels of the analyzed anatomical component. Considering the lack of a reliable system to establish the direct correspondence of the fractured surfaces, to align the surfaces of the bone fragments, the intervention of the operator is necessary. The software that processes the imaging data uses a segmentation process through which the images are transformed into the STL (Standard Triangle Language) file format, in a structure like 3D meshes. This technology used in the description of the surfaces can lead in certain situations to the attenuation of some anatomical details that might not accurately characterize some subtle injuries. For this reason, we preferred the use of ultra-high-resolution 3D printing technology with a precision of 2 μm.

### 2.3. Physical Preoperative Planning

Three-dimensional printing of the fractured pelvis allows us to obtain a physical model with the support of which the patient’s condition can be thoroughly explored. In step (3), creation of the pelvis physical model, the coded model in STL format is used for 3D printing, because these files mathematically describe the geometry of the three-dimensional bone surface. In stage (4), preoperative physical simulation, the physical model of the 3D-printed pelvis is used to select the dimensions and shape of the appropriate reconstruction plates. Preoperatively, in this model, the plates needed in the subsequent reduction and stabilization of the bone can be prepared. For good bone reduction and stabilization, it is necessary that the fixation plates follow the shape of the bone on which they are mounted. The pelvic bone has an overly complex shape. If the fractures occur in areas with complicated bone shapes, there is a risk that the fixation plates will not contour well enough to the patient’s bone. Thus, certain undesirable situations may occur, such as intra-articular screw penetration, mismatch in the acetabular roof, and progressive loss of fixation, leading to instability [35]. In this case, the patient needs to undergo a new surgery to remove the fixation plates and insert new ones for a better fit. 

### 2.4. Surgical Intervention and Postoperative Imaging Validation

Stage (5), orthopedic surgery, uses the study results from the preoperative simulation stage. In the first instance, the need for multiple interventions is taken into account due to the approaches, and the patient is implicitly positioned in different planes for the appropriate placement of the implants. Also, operating times can be reduced along with blood loss, and surgical decisions are improved intraoperatively. Thus, multidisciplinary teams can be prepared in advance to participate in the intervention. The chance of intraoperative neurovacuolar injuries is reduced from the start by simulating the trajectory of the screws on 3D models. The precision of their placement also helps to ensure the best stability of the construction through the efficient use of the available bone stock. Stage (6), imaging validation of the intervention, aims to evaluate the effectiveness of the surgical intervention by assessing the usefulness of the information obtained in the preoperative physical simulation stage. For this, CT images are collected, and the degree of reduction and the definitive positioning of the implants are evaluated.

The criteria for validating the theoretical model in practice consist of assessing the usefulness of the stages that make up the clinical workflow integrating 3D printing technologies in preoperative planning and performing surgery for the reduction and stabilization of pelvic fractures following a pilot surgical intervention. Later, this will be applied in the Orthopedics department on an extensive scale and its effectiveness will be evaluated. 

## 3. Results

**Figure 2:**
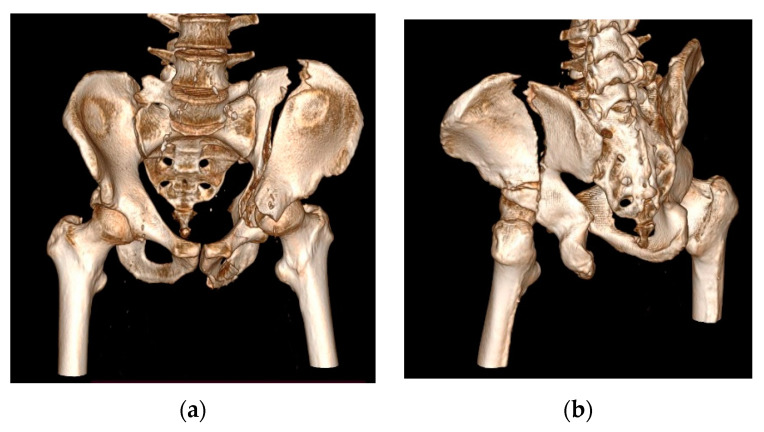
Three-dimensional reconstruction of preoperative CT imaging in RadiAnt DICOM (Digital Imaging and Communications in Medicine) for the patient included in this study: (**a**) anterior view; (**b**) postero-lateral view.

To determine the effectiveness of the theoretical model developed within the research and to ensure its practical validation, we evaluated the applicability of clinical workflow integrating 3D printing technologies in preoperative planning and performing surgery for the reduction and stabilization of pelvic fractures on a case that was presented in the continuation of this research. This is a 54-year-old man admitted to the Orthopedic Department of the Targu Mures County Emergency Clinic Hospital. He suffered a trauma by crushing, after which he was diagnosed with a both-column acetabular fracture (presenting a high anterior column fracture), which was also associated with a posterior superior wall fracture (Figure 2). The fracture was classified as AO/OTA–62C1g (Arbeitsgemeinschaft für Osteosynthesefragen/Orthopedic Trauma Association). The polytrauma whole-body CT revealed no other concomitant injuries; thus, the first patient-specific preoperative modeling phase started in the emergency room with the acquisition of the 3D Imagistics. The goal was to take advantage of the general stabilization period of the patient and use the time efficiently under 48 h. In terms of differences with traditional acetabular fracture management, we formulated an optimized and accurate personalized surgical plan before the operations that could allow us to improve the reduction’s safety and precision. 

**Figure 3:**
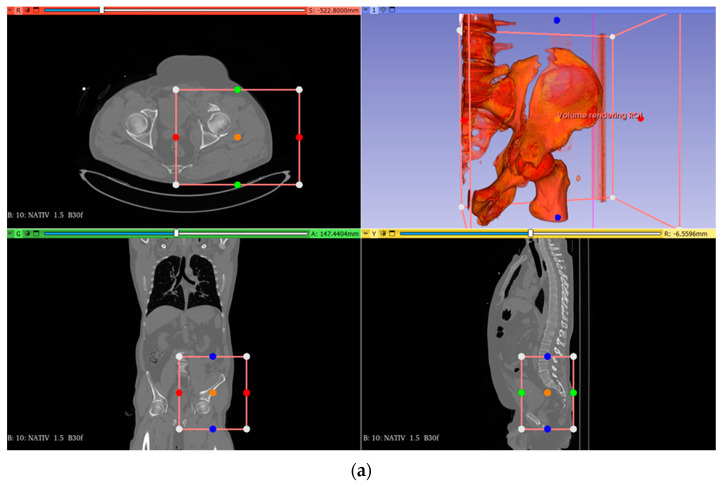

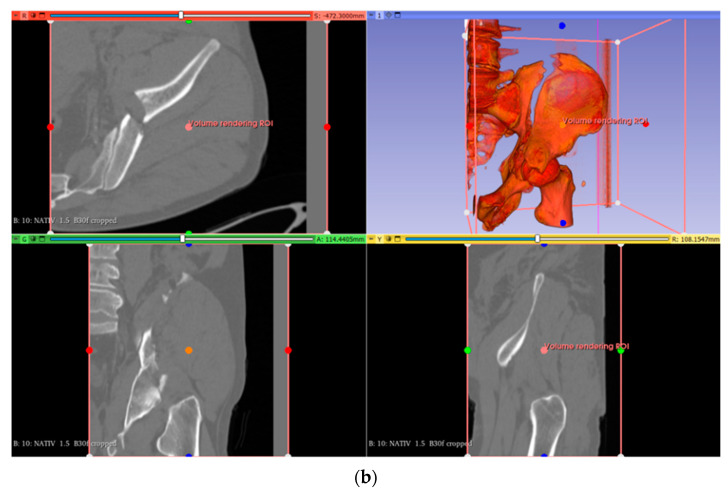
The virtual model of the acetabulum fracture: (**a**) volume rendering; (**b**) crop volume.

For segmentation and reconstruction, we used the software packages Invesalius (version 3.1.1, CTI, Sao Paulo, Brazil) [36], MeshLab (version 2023.12, ISTI—CNR, Pisa, Italy) [37], and FreeCAD (version 0.21.2, LGPL, FSF, Boston, MA, USA) [38]. Figure 3a,b show the results of the STL (Standard Triangle Language) segmentation process of the acetabular fracture, which is prepared for 3D printing. This constitutes the virtual model of the hemipelvis.

**Figure 4:**
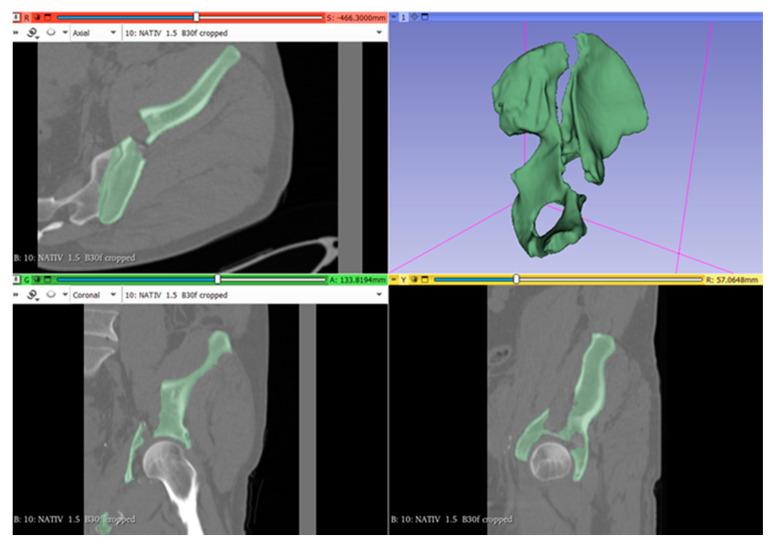
The virtual model of the acetabular fracture—editorial segment.

Analysis of bone fragmentation results in the recognition of bone segments partially detached from the supporting bone, with sizes and volumes large enough to be taken into account for clinical importance (Figure 4). This model coded in STL format is further used for 3D printing because STL files mathematically describe the geometry of the three-dimensional bone surface.

**Figure 5:**
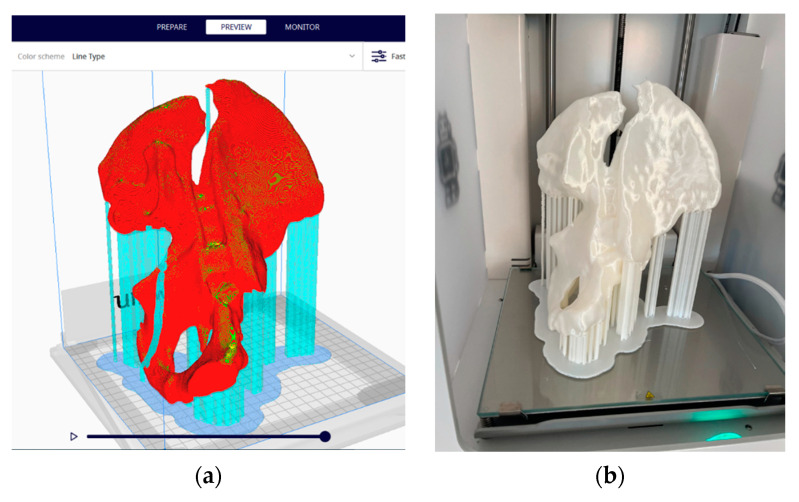
Three-dimensional printing of the acetabular fracture physical model: (**a**) preparation for printing in Ultimaker Cura software; (**b**) the actual print sustained by cork supports and platform.

The preparation of the 3D model for printing was conducted with the support of the Ultimaker Cura software [39]. The parameters used were layer thickness—0.2 mm; wall thickness—1.2 mm; bottom and top thickness—1.2 mm; filling—10%; and printing speed—50 mm/s, and the print was supported from all directions and adhered to the platform in the form of cork (Figure 5a).

Three-dimensional printing was achieved through an FDM (Fused Deposition Modeling) additive manufacturing process, which creates three-dimensional objects by thermoplastic extrusion of filaments (Figure 5b). An Ultimaker 2+ printer equipped with a 0.4 mm nozzle and a metal Poly Lactic Acid (PLA) White (Ultimaker) filament with a diameter of 2.85 mm [40] was used. Print time was 21 h and 37 min.

**Figure 6:**
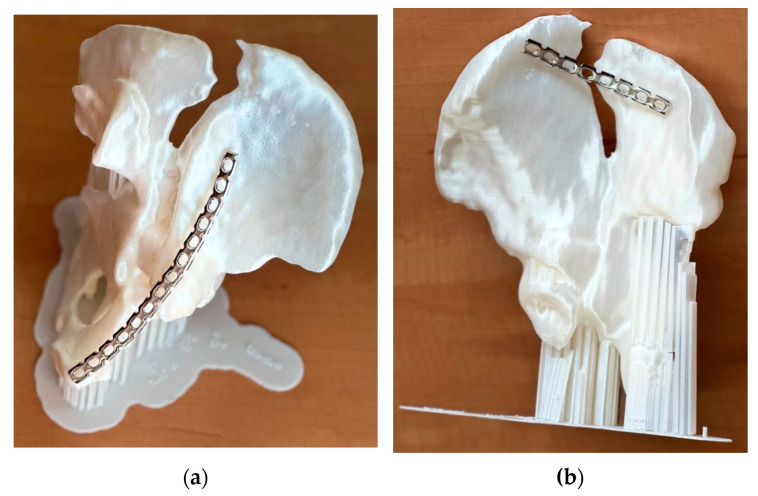
Preoperative physical simulation (image of the 3D-printed model and the plates mounted on it—before being inserted into the patient): (**a**) anterior plate view; (**b**) posterior plate view.

Visual inspection of the printed model quality reveals a good match between the radiographic images and the physical model, on which the relevant anatomical details are visible: iliac crest, ischial spine, ischial tuberosity, obturator foramen, arcuate line, and pubic tubercle. The 3D-printed model makes visual and tactile information of the affected hemipelvis available. In this complex case, the model allowed a thorough examination of the natural size of the operative field. The final decision was to perform two different interventions, to place a plate from the front and one from the back. Thus, the location of the reconstruction plates, their exact size, and their pre-modeling were chosen (Figure 6).

In the first intervention, a left ilio-inguinal approach was practiced with an arched incision above the iliac crest and preparation of the muscle planes. A retroperitoneal approach was performed, and the peritoneal sac was moved away cranially. At the time of evacuation of the retroperitoneal hematoma, a lesion was revealed at the level of the left external iliac vein. Therefore, the left iliac pedicle was assured, and the left iliac vein was sutured. Later, the medial and lateral windows were prepared at the level of fractured sites by dislocating the iliopsoas muscle. The fractured fragments were reduced by orthopedic maneuvers and osteosynthesis was performed with a premolded 14-hole reconstruction plate, three screws at the level of the ilio-pubic ramus, and six screws at the level of the iliac crest. 

The second intervention took place after 4 days and a left posterior iliac approach was performed, including disinsertion of the gluteus maximus muscle from the posterior third of the iliac wing, reduction in the left iliac wing fracture through orthopedic maneuvers, and ostesynthesis with a premolded eight-hole reconstruction plate with six screws. 

**Figure 7:**
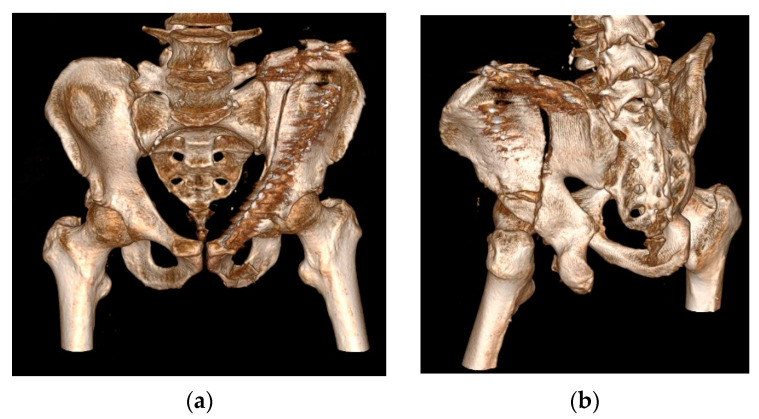
Imaging validation of the intervention—3D reconstruction of postoperative CT imaging in RadiAnt DICOM (Digital Imaging and Communications in Medicine) for the patient included in this study: (**a**) anterior view; (**b**) postero-lateral view.

The outcome of the case was satisfactory, with the rehabilitation being completed in a designated discharge unit. Postoperative antibiotic therapy was continued and completed with Trimethoprim/Sulfamethoxazole (TMP/SMX). The medical team found general satisfaction with the use of the life-size hemipelvis model. Among the advantages obtained are the precise anatomical investigation, improved preoperative planning, and the shortening of the duration of the intervention simultaneously with the increase in its precision. We appreciate that in this way, the hospital’s resources were used more efficiently. Postoperative CT imaging reveals a qualitative reduction in the fractured fragments and a satisfactory placement of the implants (Figure 7).

## 4. Discussion

As shown in the bibliometric study carried out by Li et al. [41], additive manufacturing technology is increasingly applied in pelvic surgery, and our research is part of this trend of great scientific topicality. In addition to the current state of knowledge, our research systematizes and indicates in detail the way in which 3D printing technologies are integrated into preoperative planning and performing surgery for the reduction and stabilization of complex acetabular fractures. Preoperative evaluation of pelvic fractures, especially those with displacement greater than 2 mm, which have indications for open reduction and internal fixation is notoriously difficult due to the complex anatomy of the pelvis. 

Physical models reproduce the anatomy in life size, thus enabling accurate surgical simulation. In this way, the fixation plates can be dimensioned preoperatively, and their positioning and the screw trajectory can be optimized. Our 3D-printed anatomical model allowed the preoperative simulated surgical procedure to be performed. It was useful preoperatively for determining the feasibility of intervention, and to decide the type, size, and position of fixation plates in a complex case of acetabular fracture. We found that operative and anesthetic time was reduced due to prior testing of the positioning of the fixation plates. Three-dimensional printing technology allows the manufacture of fixation plates, implants, or prostheses in less than 24 h. In cases of complex fractures, it is difficult to fit patients with standardized fixation or prosthetic systems. Our opinion is consistent with the scientific literature that in cases of complicated interventions by using 3D Physical Modeling, an improved result is obtained [42]. In addition, the risks related to successive surgical interventions of repeated insertion and removal of the fixation plates disappear.

The literature [4,43] demonstrates the utility of 3D-printed physical models, which in combination with conventional imaging results in a better understanding of the anatomy of complicated fractures, compared to the simple use of 2D and 3D images. High-quality 3D imaging has the potential to improve surgeons’ understanding when evaluating fractures. However, viewing on 2D screens induces some limitations that prevent the true benefits of 3D images from being used to their true potential. Due to the complex anatomy of the pelvic ring, it is necessary to obtain multiple images oriented in different planes for adequate preoperative planning. Image quality can be compromised by factors such as positional peculiarities of the patient’s body, intestinal gas, abdominal compression, contrast dye, and non-standardized language between the surgeon and the radiologic technician [44]. Our findings agree with the research conducted by [45] Lim et al., demonstrating that 3D physical models improved the accuracy of pelvic fracture identification. The models are also a valuable adjunct in the education of orthopedic residents, who have a valuable tool. With the support of 3D printing technology, physical models of the actual bone anatomy of the patient can be obtained, which, through visual examination, allow a better and easier assessment of pelvic deformities. The operating team can preoperatively examine the physical model that reproduces the internal structure in 3D life size, which can be touched, rotated, and examined from different positions. As a result, in cases of complicated fractures, surgeons can perform thorough assessments that allow them to make appropriate preoperative classifications and plans. In this way, the patient can be treated with improved surgical precision.

In our study, we reached similar conclusions to Huang et al. [46], showing that 3D printing technology helps orthopedic surgeons shorten the duration of surgery, reduce intraoperative complications, and improve clinical outcomes. Preoperative simulation shortens operating and instrumentation time and reduces blood loss, blood transfusion, and intraoperative fluoroscopy time. Concomitantly postoperatively, the quality of fracture reduction is improved, reducing bone union time, pain, accuracy, and functionality. However, compared to other research [46], in our study, we highlighted the printing duration of the physical model of 22 h, which can be an impediment in cases requiring emergency interventions. Djaja et al. [1] showed that when performing pelvic surgery in a limited intensive care setting, it is vital to minimize soft tissue damage, reduce intraoperative blood loss, and minimize the duration of surgery. These goals were achieved in our intervention. Additionally, we appreciate that using 3D printing in the case studied by us did not increase the risk of complications. This finding is in accordance with the review conducted by Wong et al. [47], who included a total of 932 cases. In terms of geographical regions, preoperative planning using 3D printing technologies in the field of traumatology has been certified in Asia, Europe, and North America based on 2328 cases, but an international consensus is still lacking regarding a well-defined clinical workflow [48]. Like our case study findings, other study findings [49,50] indicate that among patients who underwent open reduction and internal fixation for pelvic fractures, there was no significant difference in length of hospital stay associated with 3D printing assistance versus without it.

This study, in which we described our experience of successfully using a 3D-printed physical model in a complex case of pelvic fracture, also has some limitations. Although our results are encouraging, the number of cases indicated by the scientific literature is limited [20]. The use of the 3D-printed physical model gave us professional satisfaction, but we did not have a system of indicators that would allow us to objectively compare with other complex interventions that did not use 3D-printed models in preoperative planning. Although 3D printing technology is continuously improving, becoming widely accessible and low-cost, there are a number of regulatory, security, and safety barriers to its widespread application in orthopedic surgery. Other limitations come from long printing durations that might not be suitable for emergency interventions. The software and hardware learning curve is a process that is improved over time due to learning and increased proficiency of the specialized staff. The requirement is to have mixed teams of surgeons and medical engineers. The estimate is that by employing three times the clinical workflow integrating 3D printing technologies in preoperative planning and performing surgery for the reduction and stabilization of pelvic fractures, it can be applied with a lot of professionalism. The time durations can be higher at the beginning (up to 40 h per case), but over time, they can decrease to 22 h per case, or even less with the evolution of printing technologies. The 3D model is made of PLA, which is a biodegradable, biocompatible, and non-toxic material. It is used in the medical domains for scaffolding, orthopedic implants, drug delivery systems, and tissue engineering.

From these limitations, we have identified a series of research directions. Through further research, a system of indicators could be developed that would allow the comparison of preoperatively planned interventions with the support of 3D-printed physical models and those that are classically planned. They can be included in systems for evaluating labor practices and sustainability in healthcare [51,52]. Research into security, safety, and regulatory aspects would facilitate the widespread expansion of the technology in orthopedic surgery [53]. Other research directions consist of the construction of complex geometric models that enable patient-specific devices based on preoperative imaging, but also the development of complex porous guides that increase the stability of implants [54]. Also, future research must be oriented towards the improvement of printing technologies and the materials used to facilitate the shortening of printing durations so that the developed methodology can be used on a large scale in emergency situations. In the future, for this type of surgical intervention, larger prospective study cohorts can be formed to follow the elaborated clinical workflow and to highlight complications and favorable results with a reasonable follow-up.

## 5. Conclusions

The clinical workflow developed in this research constitutes a valuable tool that integrates 3D printing technologies into surgical practice for the reduction and stabilization of complex acetabular fractures. It deals with the preoperative, intraoperative, and postoperative periods, indicating through the sequence of six stages the way in which new three-dimensional technologies contribute to increasing the efficiency of surgical interventions. The practical validation of the theoretical concepts was conducted on a case of pelvic fracture for which we developed a 3D-printed physical model. With its support, we planned the surgery, which was evaluated and managed effectively with improved surgical precision and reduced complications. In the case studied, the accuracy and cost-effectiveness of this technique were remarkable.

## Data Availability

The data used in this study can be requested from the corresponding author.
